# Neonatal epididymo-orchitis caused by *Salmonella*: A case of successful non-surgical management

**DOI:** 10.1016/j.eucr.2024.102886

**Published:** 2024-11-15

**Authors:** Avaneesh Kunta, Samantha Gibson, Abhishek Seth, Kenneth A. Alexander, Adriana Cadilla, Pamela Ellsworth

**Affiliations:** a University of Central Florida College of Medicine, Orlando, FL, USA; b Division of Pediatric Urology, Nemours Children's Hospital, Orlando, FL, USA; c Division of Infectious Diseases, Nemours Children's Hospital, Orlando, FL, USA

## Abstract

Neonatal epididymo-orchitis (EO) is a rare condition, particularly when caused by S*almonella*, a pathogen typically associated with gastroenteritis. We present the case of a 15-day-old male who developed right-sided scrotal swelling and erythema. Scrotal ultrasound confirmed EO without signs of torsion or abscess. Further investigation revealed potential *Salmonella* exposure from feeding bottles, and stool PCR confirmed the diagnosis. The patient responded well to antibiotics, avoiding surgical intervention. This case highlights the rarity of *Salmonella* EO in neonates and the value of early imaging, thorough history-taking, and environmental exposure assessment in guiding conservative management and avoiding surgery.

## 1 Introduction

Acute scrotal swelling in neonates presents a diagnostic challenge due to its broad differential, ranging from benign conditions to surgical emergencies like testicular torsion. Among the rarer causes of acute scrotum in infants is epididymo-orchitis (EO), especially when it occurs secondary to an uncommon pathogen such as *Salmonella*. While most *Salmonella* infections manifest as gastroenteritis, extraintestinal involvement such as in the genitourinary tract, is rare but documented in the pediatric population.^1^, ^2^, ^3^ Neonatal EO remains an uncommon presentation, with only a few cases of *Salmonella*-related infections reported in the literature.^4^, ^5^, ^6^, ^7^, ^8^, ^9^, ^10^, ^11^

In this report, we present a unique case of *Salmonella* EO in a 15-day-old male infant. This case highlights the importance of non-invasive diagnostics, thorough history-taking, and the need to consider uncommon infectious etiologies in the differential diagnosis of neonatal scrotal swelling to prevent potential unnecessary surgical intervention.

## 2 Case presentation

**Fig. 1:**
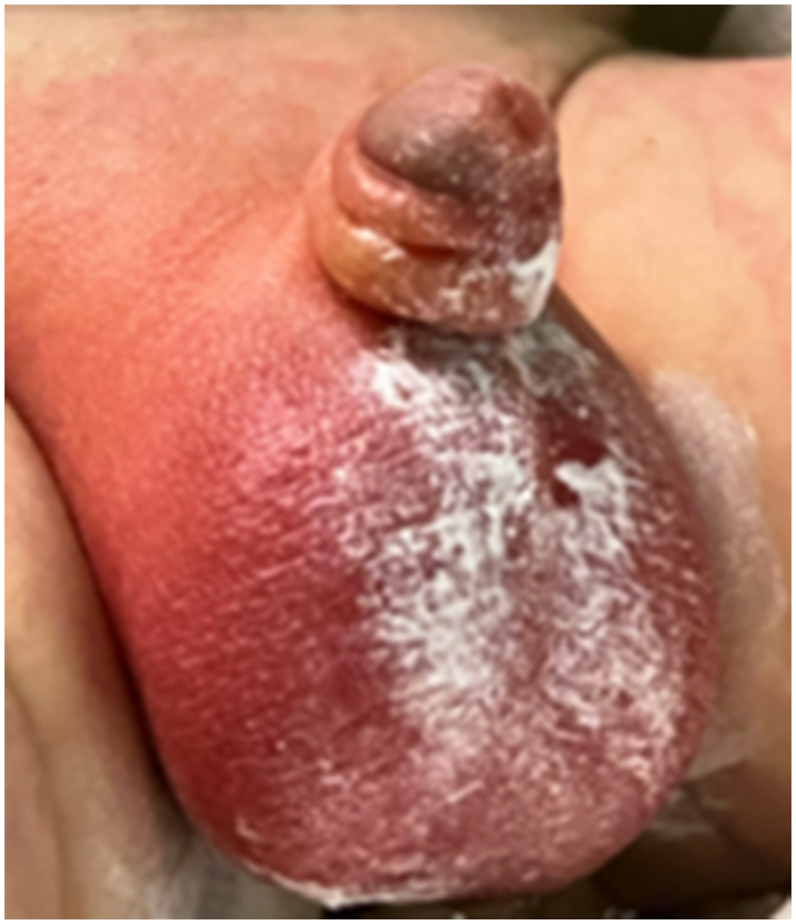
Initial Scrotal Swelling and Erythema on Gross Examination in Emergency Department

A 15-day-old, 3.7-kg term male, born via cesarean section due to maternal polyhydramnios, presented to the emergency department with a one-day history of right-sided scrotal swelling, redness, and tenderness (Fig. 1). The parents initially noted a red rash on the scrotum, which they treated with topical diaper rash cream; however, the symptoms progressed, with increased scrotal swelling and erythema. They also observed fussiness during diaper changes and a firm texture to the right hemi-scrotum. The patient's neonatal course was otherwise uncomplicated, with routine vaccinations received. He was formula-fed*,* and his medical history was unremarkable, with no recent fevers, trauma, or sick contacts. In the days leading up to presentation, the parents reported irregular bowel movements, describing an increase in diaper changes, with 6-8 stools *per*24-h period. They characterized the stools as mucous diarrhea. The persistence of these abnormal bowel patterns, fussiness with diaper changes, and the lack of response to over-the-counter topical treatment ultimately led them to seek emergency care.

**Fig. 2:**
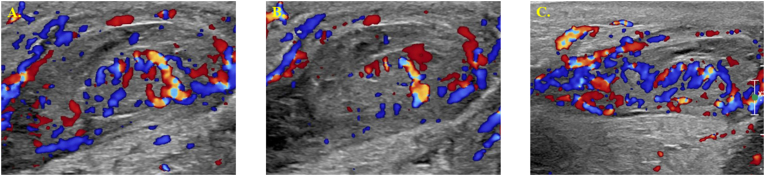
Ultrasound results on Initial ED Presentation. (A) Right Testicle, (B) Right Epididymal Head, (C) Right Inguinal Canal

On examination, the patient was afebrile and hemodynamically stable. Physical examination revealed a swollen, erythematous, indurated, and tender right hemi-scrotum. The left testicle was unremarkable except for mild hydrocele. Abdominal examination revealed a flat, non-distended abdomen with normal bowel sounds and no palpable tenderness, masses, or hernias. Scrotal ultrasound revealed right-sided EO with associated enlargement and hyperemia of the epididymis and spermatic cord, a small complex hydrocele, and thickening of the scrotal wall (Fig. 2). There was no abscess identified and no evidence of testicular torsion. Initial laboratory tests showed a white blood cell count (WBC) of 13.8 with neutrophil predominance (62.1 %) and elevated inflammatory markers, including procalcitonin (3.83) and C-reactive protein (1.3). Urinalysis revealed only trace protein and was otherwise within normal limits. Given the negative urinalysis, a renal and bladder ultrasound was performed to rule out congenital abnormalities that could cause chemical epididymitis, which returned normal results.

In the context of evaluating for late-onset sepsis, the patient underwent a blood culture, urine culture, and lumbar puncture with cerebrospinal fluid (CSF) cultures. Empirical antibiotics—ampicillin and gentamicin—were initiated. Due to concern for localized cellulitis, clindamycin was also added. Subsequent CSF analysis showed no pleocytosis, and molecular testing was negative for pathogens. Given the history of suspected bacteremia and unilateral orchitis in a neonate, an infectious disease (ID) specialist recommended empirical treatment with cefepime to cover coliforms,*Pseudomonas aeruginosa,*and*Salmonella*species. Since the patient remained afebrile, hemodynamically stable, and had negative urine cultures, the ID and Urology teams decided to continue empiric antibiotics and monitor the patient rather than pursue surgical intervention. Lumbar puncture, gram stain, and a viral polymerase chain reaction (PCR) panel did not reveal any bacterial or viral source of infection.

**Fig. 3:**
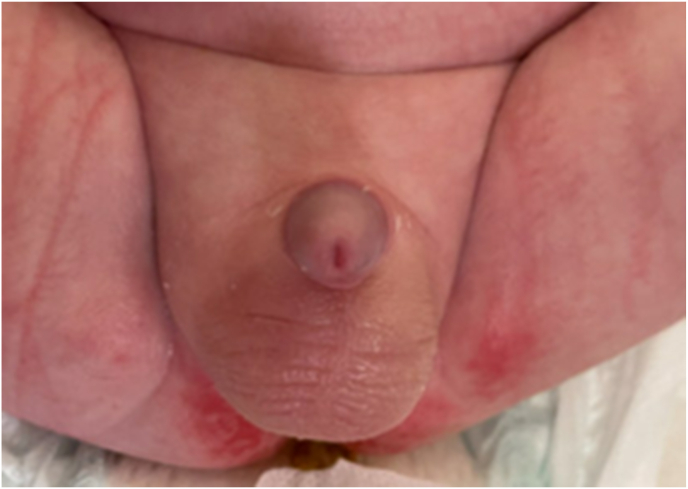
Improvement in Scrotal Swelling, Erythema, and Induration on Gross Examination at 1-Week Follow-Up

**Fig. 4:**
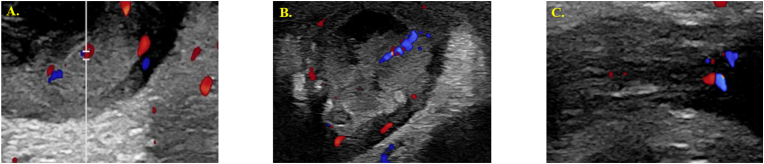
Ultrasound results on 1-Week Follow-up Visit. (A) Right Testicle, (B) Right Epididymal Head, (C) Right Inguinal Canal

The patient was admitted to the hospital for overnight monitoring and management. Overnight the patient's temperature spiked to 101.7 F briefly, but he otherwise remained afebrile. The patient continued to urinate and feed without difficulty. Repeat CBC showed improvement with WBC down trending to 10.9 and neutrophils at 52.6 %. Repeat scrotal ultrasound demonstrated normal arterial and venous blood flow and decreased scrotal skin thickening. In consultation with the ID team, further history revealed that the infant's feeding bottles had been in contact with uncooked poultry, leading to concerns about *Salmonella* cross-contamination. Given the patient's change in bowel movement frequency and quality, along with the acute febrile episode, a stool PCR test was subsequently sent and returned positive for *Salmonella species*, leading to the likely diagnosis of *Salmonella* EO. Culture results noted susceptibility to ampicillin, ciprofloxacin, and trimethoprim/sulfamethoxazole. The patient was discharged the next day following continued clinical improvement of acute hemi-scrotum and confirmation of negative blood, urine, and CSF cultures following 36 hours. Cefdinir was prescribed for 3 weeks. 1-week follow-up demonstrated resolution of scrotal erythema and induration with reduced ultrasound appearance (Fig. 3, Fig. 4).

## 3 Discussion

Our patient represents one of the youngest reported cases of neonatal *Salmonella* EO in the United States since 2005 and is, to our knowledge, the only documented case in which surgical exploration was avoided.^9^^,^^10^ A literature review identified 11 pediatric cases of *Salmonella* EO, ranging from 10 days to 14 years old, with 8 of these cases occurring in neonates between 10 days and 4 weeks of age.^1^, ^2^, ^3^, ^4^, ^5^, ^6^, ^7^, ^8^, ^9^, ^10^, ^11^ In cases like ours, where surgical intervention may be avoided, several factors should be carefully considered, including the presence of mucous diarrhea in conjunction with scrotal erythema and temporal fussiness yet normal imaging.

The patient presented with 6–8 loose, mucus-containing stools per day. Per the American Academy of Pediatrics (AAP), diarrhea is defined as a “sudden increase in the number and looseness of stools” and would classify this patient's symptoms as “Moderate” in severity.^12^ Additionally, AAP guidelines state that formula-fed infants typically pass 1–4 peanut butter-like stools daily at this age. This patient's increased frequency and loose, mucus-containing stools are therefore abnormal for his age and diet, raising concern for a possible pathological etiology.^12^^,^^13^

In the evaluation of epididymo-orchitis (EO), initial scrotal, renal, and bladder ultrasounds demonstrated good arterial and venous flow to the testis, with no signs of torsion, abscess formation, obstruction, or congenital malformations. Additionally, ultrasound revealed no upper tract or pelvic abnormalities, and urinalysis and urine culture results were negative. From a urological perspective, these findings suggested that EO was unlikely to stem from structural causes, such as an ectopic ureter or pyelonephritis, given the absence of bacteria in the urine. Consequently, both the Infectious Disease (ID) and Urology teams opted for observation rather than surgical intervention. These findings, along with negative blood, urine, and cerebrospinal fluid cultures, prompted further investigation into potential environmental exposures, ultimately leading to the discovery of cross-contamination with the neonate's feeding bottles. Stool PCR detected *Salmonella*and culture confirmed susceptibility to ampicillin, ciprofloxacin, and trimethoprim/sulfamethoxazole, which explained the patient's partial response to the empiric antibiotics.

EO is a rare complication of *Salmonella* infection in neonates, with pathogenesis often involving hematogenous spread from the gastrointestinal tract, where *Salmonella* persists following ingestion. Cross-contamination with food or water is the most common transmission route.^8^^,^^9^ In this case, the suspected source was feeding bottles exposed to uncooked chicken, a known carrier of *Salmonella*.^14^ Salmonella are classified into two main groups: those associated with enteric fever (*Salmonella*serovars Typhi, Paratyphi A, Paratyphi B, and Paratyphi C) and the non-typhoidal *Salmonella*(NTS) species.^15^ Enteric fever-associated *Salmonellae*are rare in the United States and are most often linked to international travel, which this patient had no risk factors for. The primary reservoirs for NTS include birds, mammals, reptiles, and amphibians, with transmission commonly occurring through foods of animal origin, as well as seeded fruits, vegetables, and other produce in industrialized countries. *Salmonella*can be present asymptomatically in stool, though chronic carriage is uncommon in children and more often seen in adults. NTS infections can range from asymptomatic gastrointestinal colonization to more serious conditions such as gastroenteritis, urinary tract infections, bacteremia, meningitis, brain abscesses, and osteomyelitis. Young children, especially, are more likely to present with symptoms due to a higher rate of invasive infections.^15^

Demographically, the child's age (under 5) and residence in Florida align with his diagnosis, as NTS infections are relatively common in this region.^16^ Florida reports a high incidence of invasive NTS infections, and the highest rates are observed in children under five years old (69.5 per 100,000 children).^17^ The most common NTS strains include *Salmonella Enteritidis* (22 % of cases), followed by *Salmonella Newport* (14 %) and *Salmonella Typhimurium* (13 %).

In clinical settings, laboratories differentiate enteric fever-associated *Salmonella* from NTS, but species identification typically requires molecular sequencing, which is reserved for epidemiological purposes (i.e., outbreak investigation), and is not routinely performed.^15^ Since the initial test in this case was PCR-based, it detected the presence of *Salmonella* DNA but did not determine the specific level of infection. In young children like this patient, a positive result is almost always indicative of active infection.

Ngoo et al.’s summary of case reports in addition to the most recent documented neonatal case in Feb 2019 by Trecarten et al. reveals that in all previously reported cases of neonatal *Salmonella* EO, the patients underwent surgical management.^10^^,^^11^ All patients had a salmonella species isolated from an intraoperative swab culture. These procedures were prompted by suspected testicular torsion (3/8 cases), scrotal abscess formation (2/8), incarcerated hernia (2/8 cases), and bilateral orchitis (1/9).^10^^,^^11^ All urine cultures (7/8) and CSF cultures (7/8) collected were negative. Stool cultures were obtained in 5/8 cases and returned positive results in 3 of these patients.^10^^,^^11^ Of the 2 with the negative stool culture, they each had negative blood cultures as well.

This case is notable as it represents the only documented instance in which surgical exploration was avoided in a pediatric or neonatal case of Salmonella epididymo-orchitis (EO). This outcome was achieved through careful history-taking, PCR testing for *Salmonella*, and prompt antibiotic administration. Managing general anesthesia in neonates under 30 days old presents additional challenges and risks, including the potential for postoperative apnea, which increases with earlier gestational age.^18^^,^^19^ By avoiding surgery, these anesthesia risks, along with the inherent risks of any surgical procedure, were averted. Instead, early imaging and a thorough history supported a conservative management approach. While a more conservative approach may not be the standard in adult cases, neonates have distinct physiological and developmental factors that justify a lower threshold for exploring non-surgical management in specific circumstances.^18^^,^^19^ We recommend that clinicians also consider the combination of non-invasive imaging findings, a lack of structural abnormalities, and negative culture results from sterile sites as possible criteria for initial observation and medical management in similar cases.

## CRediT authorship contribution statement

**Avaneesh Kunta:** Writing – review & editing, Writing – original draft, Supervision, Investigation, Conceptualization. **Samantha Gibson:** Writing – review & editing, Writing – original draft. **Abhishek Seth:** Writing – review & editing, Writing – original draft. **Kenneth A. Alexander:** Writing – review & editing, Writing – original draft, Conceptualization. **Adriana Cadilla:** Writing – review & editing, Writing – original draft. **Pamela Ellsworth:** Writing – review & editing, Writing – original draft, Supervision, Resources, Investigation, Conceptualization.

## 4 Financial disclosures

The authors declare that they have no known competing financial interests or personal relationships that could have appeared to influence the work reported in this paper.
